# Deep Feature Selection and Causal Analysis of Alzheimer’s Disease

**DOI:** 10.3389/fnins.2019.01198

**Published:** 2019-11-15

**Authors:** Yuanyuan Liu, Zhouxuan Li, Qiyang Ge, Nan Lin, Momiao Xiong

**Affiliations:** Department of Biostatistics and Data Science, School of Public Health, The University of Texas Health Science Center, Houston, TX, United States

**Keywords:** Alzheimer’s disease, diffusion tensor imaging images, deep learning, causal inference, feature selection, genetic-imaging data analysis

## Abstract

Deep convolutional neural networks (DCNNs) have achieved great success for image classification in medical research. Deep learning with brain imaging is the imaging method of choice for the diagnosis and prediction of Alzheimer’s disease (AD). However, it is also well known that DCNNs are “black boxes” owing to their low interpretability to humans. The lack of transparency of deep learning compromises its application to the prediction and mechanism investigation in AD. To overcome this limitation, we develop a novel general framework that integrates deep leaning, feature selection, causal inference, and genetic-imaging data analysis for predicting and understanding AD. The proposed algorithm not only improves the prediction accuracy but also identifies the brain regions underlying the development of AD and causal paths from genetic variants to AD via image mediation. The proposed algorithm is applied to the Alzheimer’s Disease Neuroimaging Initiative (ADNI) dataset with diffusion tensor imaging (DTI) in 151 subjects (51 AD and 100 non-AD) who were measured at four time points of baseline, 6 months, 12 months, and 24 months. The algorithm identified brain regions underlying AD consisting of the temporal lobes (including the hippocampus) and the ventricular system.

## Introduction

Alzheimer’s disease (AD) causes progressive brain atrophy and memory loss, is a progressive, irreversible degenerative disease of the brain, and is the most common neurodegenerative disease in the world (Struyfs et al., 2015; Zhuang et al., 2017; Liu et al., 2018a,b). AD is an increasingly prevalent disease affecting an estimated 5.4 million Americans and more than 30 million people in the world. It is estimated that these numbers will be tripled by 2050. AD is the sixth leading cause of death in the United States (Alzheimer’s Association, 2016; Leandrou et al., 2018).

Diagnosis and prediction of AD via clinical and psychometric assessments are challenging (Leandrou et al., 2018). The AD patients cannot obtain early and accurate diagnosis through clinical dementia rating and cognitive tests. A final diagnosis of AD is confirmed by histological examination at postmortem biopsy. However, the histological examination of the brain for the living patients is infeasible. Individually varying brain structure, function, and pathological effects can be measured by images. Therefore, imaging plays an important role in improving diagnosis and prediction of AD. According to the recommendation by the National Institute of Neurological and Communicative Disorders and Stroke–AD and Related Disorders Association (NINCDS-ADRDA) Work Group, the clinical classification of AD should explore the image markers: magnetic resonance imaging (MRI), diffusion tensor imaging (DTI), positron emission tomography (PET), amyloid-PET, tau-PET, and abnormal neuronal cerebrospinal fluid (CSF) markers (tau and/or Aβ) (Dubois et al., 2007; Leandrou et al., 2018; Liu et al., 2018a,b).

As the size of the imaging datasets increases, manual analysis of imaging data is tedious and time-consuming. Computer-aided diagnosis (CAD) of AD that combines computational models and analytical tools for high-dimensional imaging data analysis is emerging as one of the major tools for diagnosis and prediction of AD (Dimitriadis et al., 2018; Leandrou et al., 2018). The widely used machine learning (ML) methods in CAD include discriminant analysis (DA), logistic regression (LR), random forest, neural networks, and support vector machine (SVM) (Lorenzi et al., 2017; Sarica et al., 2017; Dimitriadis et al., 2018; Leandrou et al., 2018). Deep learning, a rapidly resurging subfield of ML, outperforms many classical ML approaches and is emerging as a major analytic platform in ML (Esteva et al., 2019). Deep learning with massive amounts of computational power has produced a revolution in driverless cars, speech recognition, and imaging analysis (Waldrop, 2019) and demonstrated great potential for the diagnosis and predictive power in tuberculosis (Heo et al., 2019), cancer (Esteva et al., 2017; Haenssle et al., 2018; Ghatwary et al., 2019; Ladefoged et al., 2019), diabetic retinopathy (Gulshan et al., 2016), chronic kidney disease (Ravizza et al., 2019), AD (Payan and Montana, 2015; Hosseini-Asl et al., 2016; Sarraf and Tofighi, 2016; Ju et al., 2017; Ding et al., 2018; Wada et al., 2018; Spasov et al., 2019), and conversion from mild cognitive impairment (MCI) to AD (Choi et al., 2018; Spasov et al., 2019). There is a growing interest in the application of deep learning to health care and medicine.

Despite its great progresses in computer vision, natural language processing, control, decision making, diagnosis, and early detection of complex diseases, deep leaning is also well known as a “black box” owing to its low interpretability to humans and still has a serious opacity problem (Waldrop, 2019). Overcoming the limitation of the lack of transparency and interpretation remains a great challenge for deep learning (Dubois et al., 2007). In this paper, we develop a novel general framework that integrates deep leaning and causal inference for image classification. The new framework for image analysis consists of two stages: (1) develop convolutional neural networks (CNN) to classify AD status on the basis of DTI and use of occlusion map to find image regions that are most distinctive for disease status and (2) the use of state-of-the-art causal inference tools to determine if the selected image regions are causal for AD.

Brain anatomy, structural connectivity, and physical connection between brain regions that are characterized through water molecular diffusing within white matter tracts can be measured by DTI. The imaging signals provide intermediate endophenotypes. Genetic variants will influence brain microstructure, function, and disease development. Understanding the role that genetics has in imaging and disease variation is key to understanding the causal chain of complex diseases (Jahanshad et al., 2013; Bycroft et al., 2018; Elliott et al., 2018). Therefore, to further cover the genetic bases of brain structures and function, and mechanism of AD, a joint analysis of the genetic brain images and AD will be carried out. We will assess both association and causal relationships among genetic variants, brain regions, and AD.

## Materials and Methods

### Materials

The DTI images used in this study are downloaded from the Alzheimer’s Disease Neuroimaging Initiative (ADNI); the size of each image was 91 × 109 × 91. ADNI is a longitudinal multicenter study designed to develop clinical, imaging, genetic, and biomedical biomarkers for the early detection and tracking of AD^1^ (Alzheimer’s Disease Neuroimaging Initiative, 2019). DTI images were recorded for every participant from different time points in which they joined the research study. The diagnostic results were normal control (NC), MCI, and AD. In this study, DTI images of 151 individuals from NCs (100 images) and AD (51 images) groups were chosen from four different diagnostic time points: baseline, 6 months, 12 months, and 24 months.

### Image Preprocessing

To make sure that all the images for this analysis are comparable, we register all the DTI image data for every subject at every time point to the common template, which can be downloaded from the McConnell Brain Imaging Centre^2^. We utilized a strategy of combination of linear and non-linear registration algorithm to map each individual DTI data to the common template. During the linear image registration procedure, we first map the image data to the common template to make sure all the images are within the standard brain region by using FLIRT (FMRIB’s Linear Image Registration Tool) from FSL (FMRIB software library) image analysis suite^3^. Then we further applied non-linear registration algorithm, which is implemented in RNiftyReg to map the image details within the standard brain. The linear image registration process helps us restrain each individual DTI image to a standard template, and the non-linear image registration helps us to make sure that the registered image maintains the structures details as the original data.

### Genetic Data Preprocessing

We performed quality control (QC) in both individual level and single-nucleotide polymorphism (SNP) level QC in the plink binary format. For the individual level QC, the following steps were applied to the data:

1. Individuals with discordant gender information were removed from the data.
2. Individuals with missing rate >10% were removed from the data.
3. Individuals with heterozygosity rate of more than three standard deviations from the mean were excluded from the data.
4. Individuals with identity by descent (IBD) > 0.185 were excluded from the data.

After the individual level QC was conducted, the following steps for SNP level QC were further applied to the data:

1. SNPs with missing genotype rate >10% were excluded from the data being analyzed.
2. SNPs with *P*-value for Hardy–Weinberg equilibrium (HWE) test <1*E*-6 were excluded from the data.
3. SNPs without polymorphism were removed from the data.

Then pre-imputation QC tool from McCarthy Groups was further applied to check the data against 1000G reference data. The imputation of the genetic data was conducted under the SHAPEIT + IMPUTE2 framework in the internal computational clusters. The 1000G reference data were used as the reference panel for imputation. After the imputation, the SNP level QC steps were applied again to the data to produce the final genetic data for analysis. Finally, a total of 1,589,061 common SNPs in 36,480 genes genotyped in 151 individuals were included in analysis.

### Architecture of Convolutional Neural Network

The CNN model Visual Geometry Group (VGG) that won the first and second places in the localization and classification tracks, respectively, in the ImageNet Challenge 2014 was chosen for image classification and prediction (Simonyan and Zisserman, 2014). To improve the classification accuracy, the VGG utilized smaller receptive window size and increased the depth of the network. Furthermore, to prevent overfitting and improve the image region recognition ability of the networks, global average pooling (GAP) layer was used as a structure regularizer and localizer in the model to identify the complete extent of the object and exactly which regions of an image are being used for classification (Zhou et al., 2016).

**FIGURE 1:**
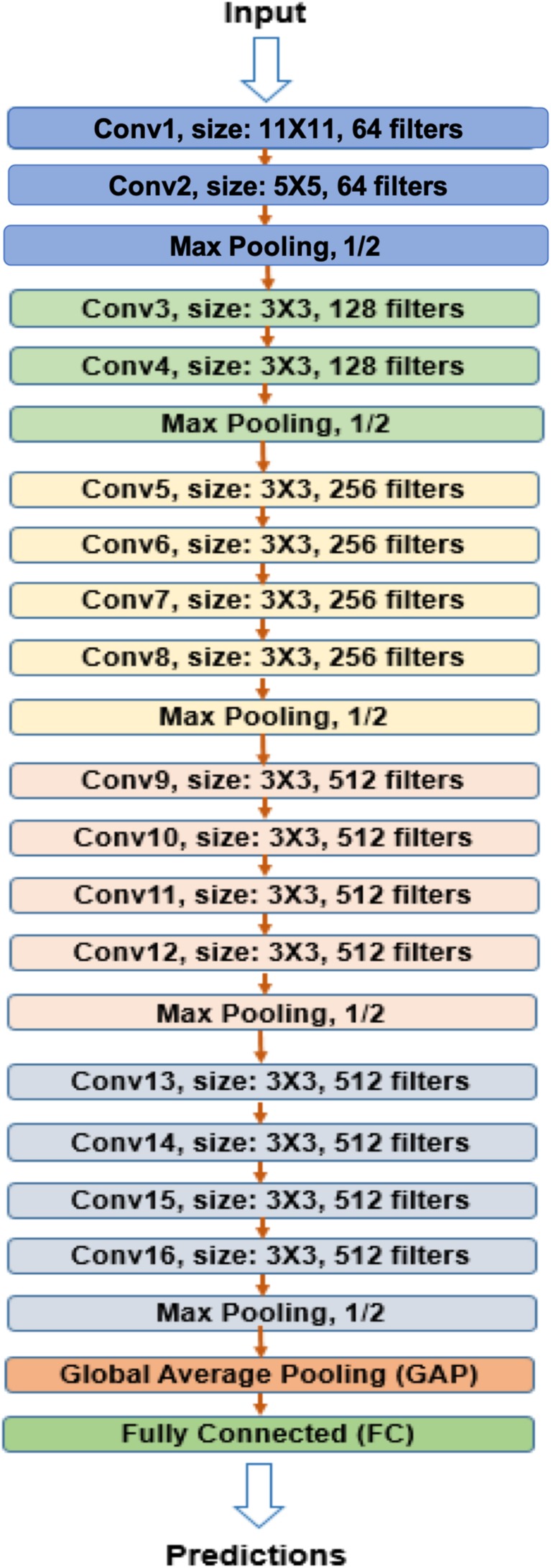
VGG-GAP model architecture. The CNNs in the model included five max pooling layers and one GAP layer before fully connected layer. VGG, Visual Geometry Group; GAP, global average pooling; CNN, convolutional neural network.

As is shown in Figure 1, the network contained five max pooling layers, followed with a GAP layer before a fully connected softmax layer with two nodes.

Three-dimensional (3D) whole brain images with 109 × 91 × 91 size were input into CNN. DTI measures microscopic random motion of water molecules, which uncovers the orientation of surrounding tissues, and provides tract information on brain structure. Convolution of an image with different filters can perform operations that capture various types of features and directional information of DTI images and can preserve tract of DTI and the relationship between pixels. 3D CNNs (3D-CNN) with five convolutional layers and three fully connected layers were used for AD prediction. A 3D filter was applied to the dataset, and the filter moves in three directions (*X*, *Y*, *Z*) to calculate the low-level feature representations. Specifically, 3D filters were arranged as in Table 1.

**TABLE 1:** 3D filters in five convolutional layers.

**Conv layer**	**Filter size**	**Stride in (*X*, *Y*) direction**	**Stride in *Z* direction**
Conv 1	11 × 11 × 11	4	4
Conv 2	5 × 5 × 5	1	1
Conv 3	3 × 3 × 3	1	1
Conv 4	3 × 3 × 3	1	1
Conv 5	3 × 3 × 3	1	1

*3D, three-dimensional.*

To overcome the small sample size limitation of medical images, image augmentation techniques were used (Aderghal et al., 2017). The first technique we applied was Gaussian filters to blur the image to mimic the possible variations in the original images. A filter size of 3 × 3, 5× 5, and 7× 7 were used with spread parameters of 0.7, 0.7, and 0.6, respectively. The second augmentation technique we used was translation, where we shifted the images by ±1 pixel in each dimension. This imitates the possible variations in registration process where the images were aligned with the template. Finally yet importantly, the images were flipped horizontally because some regions of the brain (e.g., the hippocampus) are symmetrical to enlarge our sample size. To balance the data, we randomly duplicated some images from the under-sampled category. Data augmentation and class balancing produced over 20 times more data than the original dataset.

The model was trained in the Texas Advanced Computing Center (TACC) Maverick2 with NVIDIA GTX 1080 Ti GPUs.

### Deep Feature Selection for Diffusion Tensor Imaging Images

Prediction difference analysis for visualizing the response of CNN to a specific input was used to select features for DTI image classification (Zintgraf et al., 2017). Specifically, prediction difference analysis estimates the importance of input pixels by calculating the effect of removing information from the imaging on the class prediction precision (Zeiler et al., 2014).

A sliding window (patch) of 3 × 3 × 3 was applied to each image. The imaging signals contained in the sliding window were taken as a feature. Each one 3 × 3 × 3 patch was replaced by randomly sampled values from multivariate normal distributions. The resulting new image where the imaging feature (information) was removed was input into a previously trained CNN model to obtain probability *p*_1_ for predicting AD. Let *p*_0_ be the probability of predicting AD using the original images [without removing the feature (information)]. The relative importance of the feature was evaluated by Zintgraf et al. (2017).

(1) $$d=\log\left(\frac{\frac{p_{0}}{1-p_{0}}}{\frac{p_{1}}{1-p_{1}}}\right)$$

The sliding window moved across the entire image and a relevance matrix, *W* of the same size as the whole image was generated, which reflected the relevance importance of all image pixels. A positive value indicated that the pixel contributed evidence for the classification of AD, whereas a negative value showed that the pixel contributed against the classification of AD. For details, please see Zintgraf et al. (2017).

### Conditional Generative Adversarial Network and Classifier Two-Sample Tests for Causal Discovery

**FIGURE 2:**
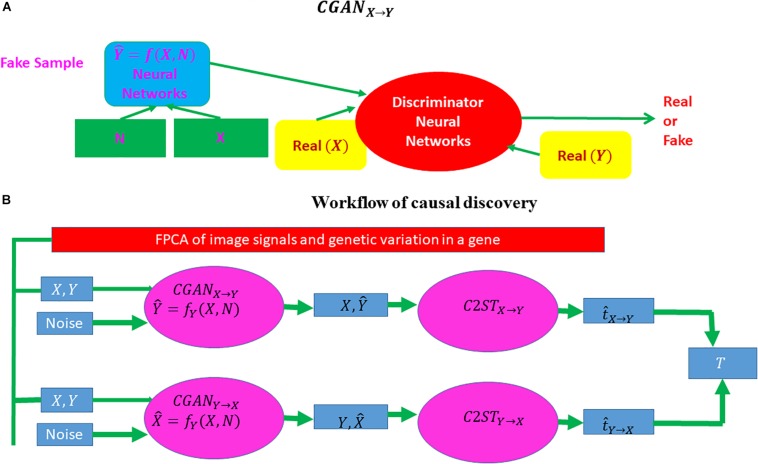
Workflow of causal inference using CGAN and a classifier two-sample test. CGAN, conditional generative adversarial network. **(A)** A visual explanation of CGAN and **(B)** the complete workflow of causal discovery.

Three-dimensional functional principal component (FPC) scores were used to summarize the imaging signal information of the brain region (Xiong, 2018). Similarly, 1D FPCs can be used to summarize genetic information in the gene. Conditional generative adversarial networks (CGANs) will be used to discover causal relationships between the brain neuroimaging region and AD and causal relationships between the brain neuroimaging region and gene as well (Goodfellow et al., 2014; Lopez-Paz and Oquab, 2017) (Figure 2). Specifically, consider two variables *X* and *Y*, which can be binary disease status or continuous FPCs summarizing imaging signals in the brain region or genetic variation in the gene. If *X* causes *Y*, denoted by *X* → *Y*, then we have

*Y* = *f*_*Y*_(*X*, *N*_*Y*_),

where *f*_*Y*_ is a non-linear function and realized by CGAN where a neural network is used to approximate the non-linear function *f*_*Y*_(*X*, *N*_*Y*_), and *N*_*Y*_ is a noise random variable and is independent of cause *X*. Similarly, if *Y* causes *X*(*Y→X*), then we have

*X* = *f*_*X*_(*X*, *N*_*X*_),

where *f*_*X*_ is a non-linear function and *N*_*X*_ is a noise random variable and is independent of cause *Y*. Assume that *n* subjects are sampled.

We define dataset *D*_*w*_ = {*u*_*i*_, *v*_*i*_, *i* = 1, …, *n*}. We assign label 0 to dataset *D*_*u*_ = {*u*_*i*_, *i* = 1, …, *n*} and 1 to dataset *D*_*v*_ = {*v*_*i*_, *i* = 1, …, *n*}. Let *P* be the distribution of *u*_*i*_, *i* = 1, …, *n* and *Q* be the distribution of *v*_*i*_, *i* = 1, …, *n*. We use the *K nearest neighbor* (*KNN*) as a binary classifier to classify two datasets and define the test statistic *t* as the classification accuracy to test the null hypothesis of equal distributions of two datasets *P* = *Q*. Let *z* be a random variable.

The procedures for bivariate causal discovery using CGAN are summarized as follows (Lopez-Paz and Oquab, 2017):

1. Use a CGAN from *X→Y* to generate the dataset *D*_*X* → *Y*_   =  { (*x*_*i*_,${\hat{y}}_{i}$   =  *f*_*y*_ (*x*_*i*_,*z*_*i*_)),  i  =  1,…,n}.

2. Use a CGAN from *Y→X* to generate the dataset *D*_*Y→ X*_   =  {(${\hat{x}}_{i}$   =  f_*X*_ (y_*i*_,z_*i*_),y_*i*_),  i  =  1,…,n}.

3. Divide the total samples into training samples and test samples.

4. Classify two datasets : *D*_*u*_ = *D*_*y*_ = {*y*_*i*_, *i* = 1, …, *n*} versus *D*_*v*_   =  D_*X* → *Y*_   =  {${\hat{y}}_{i}$,    i=  1,…,n} and calculate the two-sample statistic ${\hat{t}}_{X\to Y}$.

5. Classify two datasets: *D*_*u*_ = *D*_*x*_ = {*x*_*i*_, *i* = 1, …, *n*} versus *D*_*v*_   =  *D*_*Y* → *X*_   =  {${\hat{x}}_{i}$,    i=  1,…,n} and calculate the two-sample statistic ${\hat{t}}_{Y\to X}$.

6. Calculate the test statistic T  =  ${\hat{t}}_{X\to Y}$$-{\hat{t}}_{Y\to X}$. Under the null hypothesis of no causal relationship or test inconclusive, the statistic *T* is asymptotically distributed as

*N*(0,σ^2^), where $\sigma^{2}=\frac{0.5}{n_{\text{test}}}-2\,c\,o\,v\,\left({\hat{t}}_{X\to Y},{\hat{t}}_{Y\to X}\right)$ and *n*_*test*_ is the number of subjects in the test set.

Association is defined as measuring the dependence or correlation between two variables and to use these dependencies for prediction that is not dealing with causal problems. Almost all currently used statistical methods in imaging genetics [such as sparse canonical correlation analysis (SCCA), sparse reduced rank regression (SRRR), and parallel independent component analysis (ICA)] are association analysis methods. These methods can detect association between genetic variation and imaging signals. It is well known that correlation or association analysis does not imply causation. The signals identified by association analysis may not have specific pathological relevance to diseases. Association signals provide limited information on the causal mechanism of diseases. Most genetic and imaging analysis questions to uncover the mechanism of the disease are causal in nature. Causation analysis is essential to the genetic analysis of complex phenotypes yet ignored for a long time.

Distinguishing causation from association is an age-old problem. Intuitively, causation implies that changes in one variable will directly make changes in the other. The essential distinction between association and causation relies on what the response will be if we intervene in the system (Lattimore and Ongv, 2018).

There are two types of causal inference: interventional causal inference and observational causal inference. Interventional causal inference learns the effect of taking an action directly via experiments, for example, randomized controlled trials. Interventional experiments are a gold standard for causal inference. However, because in human genetics we cannot change the genetic materials of human subjects, experimental interventions are unethical and infeasible. Therefore, it is essential to develop statistical methods and algorithms to predict the outcomes of an intervention from passive observation.

The additive noise models (ANMs) assume one causal direction *X* → *Y* but no reversible causal direction *Y* → *X*. Causation is asymmetric. However, the association of *X* and *Y* can be (1) *X* → *Y*, (2) *Y* → *X*, and (3) *X* → *Y*, *Y*→ *X*. Association is symmetric.

Additive noise models are based on the independence of cause and mechanism (ICM) principle. ICM assumes that causes and mechanisms are chosen independently by nature, which is a recently proposed principle for causal reasoning and causal learning (Peters et al., 2017). ICM assumes that the mechanism that generates effect from its cause contains no information about the cause, which implies that *X* and *N*_*Y*_ in the ANMs are independent. However, *X* and *N*_*Y*_ in the non-linear regression model *Y* = *f*_*Y*_(*X*, *N*_*Y*_) may be dependent.

In summary, association is studied by observed conditional distribution, and causation is investigated by interventional distribution where causal effect is determined by the effect of hypothetic manipulation of an input on an output. In other words, association is investigated by seeing, and causation is investigated by doing.

## Results

### Alzheimer’s Disease Classification and Prediction

The VGG network with 3D filters was used for classification and prediction of AD using 3D whole brain DTI images at four different time points: baseline, 6 months, 12 months, and 24 months. We consider two classes: AD and NC. AD prediction accuracy using VGG is listed in Table 2, and its sensitivity and specificity are shown in Table 3, where the first and second values in the brackets represent sensitivity and specificity, respectively. Tables 2, 3 demonstrate that the prediction accuracy, sensitivity, and specificity of VGG using the training dataset at baseline to predict AD in the test datasets at baseline, 6 months, 12 months, and 24 months were 0.8675 (0.6873, 0.9600), 0.8452 (0.6364, 0.9600), 0.8335 (0.7295, 0.8995), and 0.7463 (0.6294, 0.8853), respectively. In other cases, we can observe similar results. The area under the curve (AUC) using the training data at baseline, 6 months, 12 months, and 24 months for prediction of AD in the test datasets at the same time points was 0.8571, 0.8291, 0.8583, and 0.7756, respectively. The low sensitivity of prediction of AD may be due to small and imbalanced sample size (51 AD and 100 controls). A much higher proportion of non-AD controls have decreased sensitivity but increased specificity. Deep VGG that has a large number of parameters to be estimated requires large sample sizes. Although we used data augmentation methods to increase sample sizes, augmentation methods still did not provide large and reliable sample sizes. Large sample sizes are an important issue for increasing the prediction of accuracy.

**TABLE 2:** AD prediction accuracy on fivefold cross validation.

**Model development time point**	**Prediction time point**			
	**Baseline**	**6 months**	**12 months**	**24 months**
Baseline	0.8675	0.9123	0.8864	0.7967
6 months		0.8452	0.8963	0.7791
12 months			0.8335	0.7813
24 months				0.7643

*AD, Alzheimer’s disease.*

**TABLE 3:** Average sensitivity and specificity over fivefold cross validation.

**Model development time point**	**Prediction time point**			
	**Baseline**	**6 months**	**12 months**	**24 months**
Baseline	(0.6873, 0.9600)	(0.8073, 0.9700)	(0.7524, 0.9717)	(0.6465, 0.9313)
6 months		(0.6364, 0.9600)	(0.7778, 0.9717)	(0.5977, 0.9417)
12 months			(0.7295, 0.8995)	(0.6674, 0.8833)
24 months				(0.6294, 0.8853)

### Region Selection and Interpretation

**FIGURE 3:**
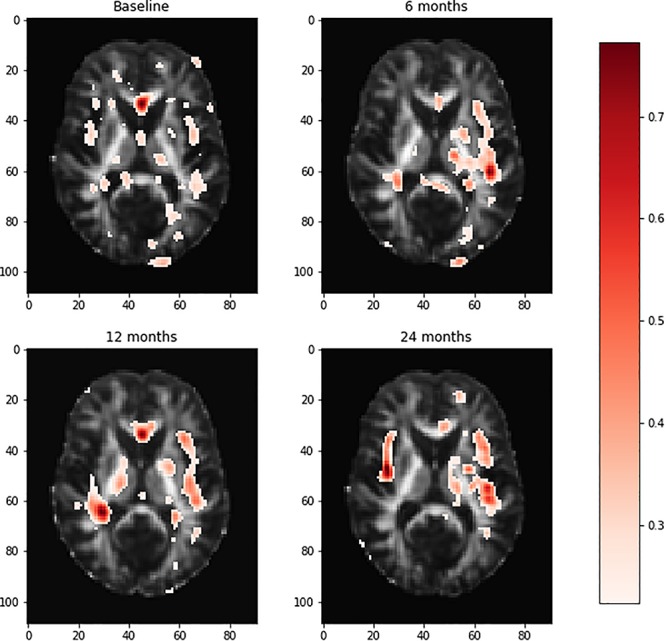
Visualization of the brain regions with relative importance values at the baseline, 6 months, 12 months, and 24 months. The deeper the red color of the brain region, the more important for AD prediction. AD, Alzheimer’s disease.

**FIGURE 4:**
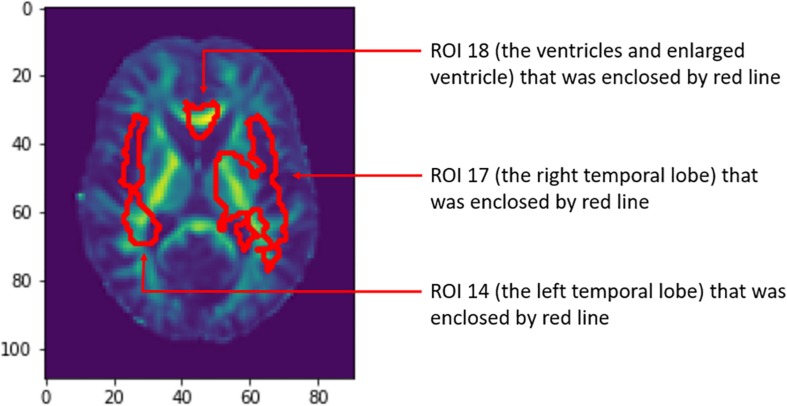
Three brain regions showed causation to AD. AD, Alzheimer’s disease.

Relative importance of value *d* was sorted. Image areas whose relative importance value was in the top 10th percentile were considered as features that contributed substantially to the prediction of AD. We identified 23 important brain regions that contributed substantially to AD prediction. The results are shown in Figure 3 where each subfigure has 91 × 109 pixel sizes, where the darker the red color is, the more important the brain region is to the prediction accuracy. The brain regions with red color included the temporal lobe (the left temporal, medial, and right temporal lobes), ventricles and enlarged ventricle, occipital lobe, and prefrontal area. To further interpret the image analysis results and increase their transparency, we tested the causal relationships between DTI image ROIs and AD disease at baseline, 6 months, 12 months, and 24 months using CGAN-based statistics. After Bonferroni correction, *P*-value < 0.0022 was the threshold to declare significance. The number of identified brain regions that showed significant causation to AD at baseline, 6 months, 12 months, and 24 months was 1, 1, 2, and 4, respectively. Table 4 lists ROIs where *P*-values for testing causation between the ROI and AD were <0.05. Three remarkable features emerged from these results. First, as time passed, AD progressed from mild (early stage), via moderate (middle stage), to severe (late stage), which resulted in atrophy of more and more brain regions. Therefore, we observed the increased number of significant causal brain regions with AD as the study time of AD increased from the baseline to 24 months. Second, in general, as AD progressed, the significance of causation between the brain region and AD increased (*P*-values for testing causation decreased). Third, the brain region in ROI 18 (the ventricles and enlarged ventricle) (Figure 4) showed significant causation to AD at all four time points (baseline, 6 months, 12 months, and 24 months). The brain regions in ROI 14 (the left temporal lobe) (Figure 4) showed significant causation at 12 and 24 months after Bonferroni correction. The literature reports that these regions are related to AD. The left temporal lobe is involved in language and AD (Cretin et al., 2015; Flick et al., 2018; Trimmel et al., 2018), and the right temporal lobe atrophy is involved in severe impairment in emotion recognition (Everhart et al., 2015) and causes frontotemporal dementia (Gliebus, 2014), with the brain ventricles often affected AD (Ferrarini et al., 2006). Ventricle enlargement is a useful structural biomarker for the diagnosis of AD (Anandh et al., 2014).

**TABLE 4:** Causations between DTI image ROIs and AD disease status.

**Time point**	**ROI index**	***P*-value**
Baseline	2	0.0463
	18	0.0005
6 months	8	0.0182
	14	0.0108
	17	0.0155
	18	0.0010
12 months	6	0.0117
	14	0.0018
	17	0.0107
	18	<0.00005
24 months	0	0.0245
	3	0.0133
	5	0.0092
	7	0.0063
	8	0.0030
	9	0.0007
	11	0.0084
	12	0.0002
	13	0.0082
	14	<0.00005
	15	0.0098
	17	0.0239
	18	<0.00005
	19	0.0210
	21	0.0363
	22	0.0166

*ROI 14 corresponds to the left temporal lobe and 17 corresponds to the right temporal lobe. ROI 18 corresponds to ventricles and enlarged ventricle and indicates atrophy of cerebral nerve tissue, which is typical in AD patients. DTI, diffusion tensor imaging; ROI, region of interest; AD, Alzheimer’s disease.*

### Genetic Studies of Two Brain Regions

To uncover genetic architecture of brain regions, in addition to genetic-imaging association analysis, we conducted genetic-imaging causal analysis using the CGAN where imaging signals within the brain region and SNPs within the gene were summarized by 2D functional principle scores and classical functional principle scores, respectively (Lopez-Paz and Oquab, 2016). The total number of candidate genes being tested was 61. After Bonferroni correction, the *P*-value for declaring significance of both causation and association was 0.00082. We presented the results of *P*-values < 0.05 in causal analysis and association analysis of genetic variation in 61 candidate genes with two brain regions, the left temporal lobe and frontal and temporal left lobe, and the right temporal lobe as seen in Supplementary Tables S1, S2, respectively, where 61 genes were obtained from genome-wide causation studies of AD in the manuscript (Lin et al., unpublished). In Supplementary Tables S1, S2, the *P*-values in bold green denote significant causation or association after Bonferroni corrections. The majority of genes that had causal or association relationships with brain neuroimaging phenotypes were identified at all time points (baseline, 6 months, 12 months, and 24 months). We also observed that these identified genes had causal or association relationships with both the left temporal lobe and right temporal lobe regions. The identified genes *CD33*, *COBL*, and *APP* that had causal relationships with brain neuroimaging regions were confirmed multiple times in the literature (Bradshaw et al., 2013; Mez et al., 2017; Kovacs et al., 2018; Van Giau et al., 2018; Huang C.C. et al., 2019; Huang C.Y. et al., 2019). It was also reported that gene *FGF4* was involved in neurodevelopmental disorders (Grillo et al., 2014), *FRMD6* was implicated in AD (Hong et al., 2012), *Dock9* played an important role in regulation of morphological changes in hippocampal neurons (Kuramoto et al., 2009), *H3F3B* was associated with a broad schizophrenia phenotype (Manley et al., 2018), *SCYL1* was involved in cerebellar atrophy (Lenz et al., 2018), *AKAP5* played a significant role in the regulation of sympathetic nerve activities (Han et al., 2016), and *PIGC* was involved in epilepsy and intellectual disability (Edvardson et al., 2017).

## Discussion

In this paper, we presented a general artificial intelligence (AI) platform for prediction of AD using DTI images. Non-transparency could be a major challenge of deep learning for medical image analysis. To meet this challenge, we introduced three approaches to medical image interpretation: feature selection and visualization, causal analysis of neuroimaging region, and genetic-imaging analysis. Feature selection and visualization methods selected and visualized brain regions as a potential pathology of AD. Further CGAN evaluation and two-sample tests discovered potential causal relationships between the brain neuroimaging regions and AD. We observed the increased number of significant causal brain regions with AD when AD progressed. In general, as AD progressed, the significance of causation between the brain region and AD increased (*P*-values decreased). We observed that the ventricles and enlarged ventricle and the left and right temporal lobes had strong causal relationships with AD. Temporal lobes including the hippocampus are crucial in AD development at the early stages, whereas the ventricles and enlarged ventricle are a useful structural biomarker for the diagnosis of AD. Joint causal analysis of genetic and images of the left and right temporal regions using CGAN evaluation and two-sample tests mapped *CD33*, *COBL*, *FRMD6*, *APP*, and other genes to the left and right temporal brain regions.

Many findings in the paper can be confirmed in the literature. For example, both prediction analysis using deep learning and causal analysis using CGAN and a two-sample test identified the brain temporal lobe region that was involved in AD. The temporal lobe includes the hippocampus and its surrounding regions. It is well known that the temporal lobe consists of structures that are vital for long-term memory. There are numerous reports that the temporal lobe including the left, medial, and right temporal lobes are involved in AD pathology (Kakeda and Korogi, 2010; Li and Chen, 2015; Menéndez-González et al., 2015; Aggleton et al., 2016; Delgado-González et al., 2017; Pettigrew et al., 2017; Wolk et al., 2017; Jung et al., 2018; Kitchigina, 2018; Persson et al., 2018; Grajski and Bressler, 2019; Kenkhuis et al., 2019; Lam et al., 2019; Pasquini et al., 2019; Xie et al., 2019). DTI discovered the functional and structural connectivity between the medial temporal lobe (MTL) and posteromedial cortex (PMC) (Buckner et al., 2008; Pasquini et al., 2019). The MTL includes the hippocampal formation and other cortices. These regions underlie memory processing through interplay with neocortical areas from the PMC. AD-related pathological changes such as tau accumulation and amyloidβ deposition often affect the PMC and MTL regions. The functional and structural disconnections between the MTL and PMC cause the development and progression of AD.

The literature confirmed the identified pathological paths from genetic variants to AD via brain regions: *CD33* → medial temporal and hippocampus (Wang et al., 2019) → AD (Pasquini et al., 2019) and *CD33* → AD (Miles et al., 2019); *APP* → medial and lateral temporal lobe (Huang C.C. et al., 2019) → AD (Buckner et al., 2008) and *APP* → AD (Zhou et al., 2011); *SCYL1* → cerebellar atrophy (Schmidt et al., 2015) → AD (Gallo et al., 2017); and *SCYL1* → neurodegenerative disease (Schmidt et al., 2007). These provided indirect evidences of identified biomarkers for unraveling mechanism of AD.

The results in this paper are preliminary. Sample sizes need to be increased and additional datasets analyzed to replicate the results. The purpose of this paper is to stimulate further discussions regarding the great challenges we are facing in developing robust deep learning platforms that combine multiple modes of imaging tools and have high accuracy across multiple datasets and uncovering causal pathways from genetic variants to disease via brain imaging regions.

## Data Availability Statement

Publicly available datasets were analyzed in this study. This data can be found here: http://adni.loni.usc.edu/.

## Author Contributions

YL developed the software and conducted the data analysis. ZL contributed to the data preprocessing and partial writing. NL contributed to data preprocessing. QG conducted the partial data analysis. MX designed the study and wrote the manuscript.

## Disclaimer

The content is solely the responsibility of the authors and does not necessarily represent the official views of the Cancer Prevention and Research Institute of Texas.

## Conflict of Interest

The authors declare that the research was conducted in the absence of any commercial or financial relationships that could be construed as a potential conflict of interest.

## Footnotes

1 http://adni.loni.usc.edu/about/#core-container

2 http://www.bic.mni.mcgill.ca/ServicesAtlases/ICBM152NLin2009

3 http://www.fmrib.ox.ac.uk/fsl/
